# Immune monitoring technology primer: immunosequencing

**DOI:** 10.1186/s40425-015-0076-y

**Published:** 2015-06-25

**Authors:** Ilan Kirsch

**Affiliations:** Adaptive Biotechnologies, Seattle, WA USA

## Abstract

**Background:**

Profiling of the immune receptor repertoire is becoming increasingly relevant to the understanding and clinical management of cancer, autoimmunity, aging, and infectious disease.

**Findings:**

A platform technology is described that provides comprehensive immune receptor profiling.

**Conclusion:**

Immunosequencing is a platform technology that allows the enumeration, specification and quantification of each and every B-and/or T-cell in any biologic sample of interest. It is based on bias-controlled multiplex PCR and high throughput sequencing and is highly accurate, standardized, and sensitive.

## Description of the technology

The adaptive immune system generates a remarkable breadth of diversity in antigen-specific TCRs and Igs by combinatorial recombination of gene segments in lymphocytes. The TCR is composed of two peptide chains, encoded by the TCRA and TCRB or TCRG and TCRD genes, respectively. There are thus two types of T-cell receptors, αβ and γδ, that differ by the TCR heterodimer type and immune function. The antigenic specificity of T-cells is in large part determined by the amino acid sequence in the hypervariable complementarity-determining region 3 (CDR3) regions of the T-cell receptors. The existence of multiple V and J gene segments at these T-cell loci permits a large combinatorial diversity in receptor composition; and the non-templated insertion or deletion of nucleotides at the V-J, V-D, and D-J junctions further adds to the potential diversity of receptors that can be encoded. Because of the potential diversity of receptors it is highly improbable to randomly converge on the same TCRB nucleotide CDR3 sequence, effectively making each CDR3 sequence a unique tag for a T-cell clone.

**Fig. 1:**
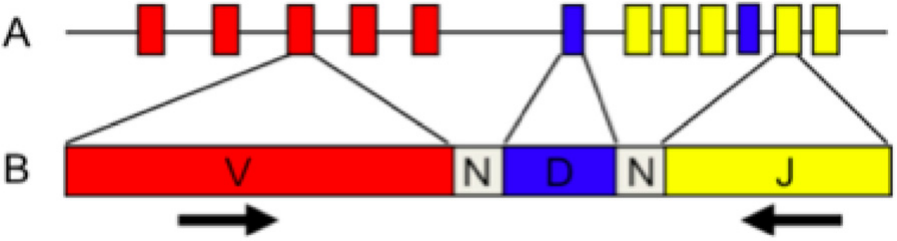
TCRB sequencing assay. **a** Multiple V, D and J segments exist in the germline genome, non-templated diversity is introduced at the junctions by insertion of random nucleotides (shown as N) **b** The assay uses a multiplex PCR with forward primers in each V segment and reverse primers in each J segment

Immunosequencing is a multiplex PCR-based based method that amplifies rearranged TCR CDR3 sequences for a given TCR locus (Fig. 1) and exploits the capacity of high-throughput sequencing (HTS) technology to characterize tens of thousands of TCR CDR3 chains simultaneously. The technology can be applied to cDNA or genomic DNA, but when it utilizes genomic DNA, the frequency of sequenced CDR3 chains is highly representative of the relative frequency of each T-cell containing CDR3 sequence in the biologic sample. Thus, this assay captures both the full TCR repertoire and specific individual clones. Given the capacity of HTS, this assay is extremely sensitive and only limited by the amount of DNA that is analyzed. Routinely, if one million cells’ worth of DNA is analyzed the assay can detect clones at sensitivity that approaches 1:1,000,000. This is about 100-fold greater than current detection methods (e.g., flow cytometry) and less subject to the occurrence of “false positive” and “false negative” results than TCR PCR. The technology provides a highly accurate and standardized method for assessment of lymphoid clonality in healthy, diseased, or malignant tissues and for identification and tracking the presence and frequency of common and rare clones within the total adaptive immune system.

## Type of data obtained/readout

For each sample, DNA is extracted and the relevant immune receptor CDR3 regions amplified and sequenced. Bias-controlled V and J gene primers are used to amplify rearranged V(D)J segments for high throughput sequencing at ~10 × coverage. Raw sequence data is uploaded and after correcting sequencing errors via a clustering algorithm, primary nucleotide sequence of the amplified regions from the immune receptors’ unique CDR3 segments are identified, quantified, and annotated according to the International ImMunoGeneTics collaboration, identifying which V, D, and J genes contributed to each rearrangement.

## Limitations of the approach

In isolation, immunosequencing only provides information relevant to the enumeration, specification, and quantification of each and every B-and/or T-cell in a given sample of interest. It can be combined with other technologies focused on immunophenotypic characterization or single cell isolation to provide more comprehensive information on the physiologic and developmental status of the individual cells or populations.

## Types of samples needed and special issues pertaining to samples

The assay is capable of providing information from fresh, frozen, or FFPE samples and has been tested on blood, bone marrow, lymph node, spleen, kidney, liver, lung, brain, ovary, skin, cell-free DNA, primary and metastatic tumor and other tissues.

## Level of evidence

Approximately 100 manuscripts and poster presentations have been published using this platform. The analytic validity, clinical validity, and clinical utility is presented in many of these publications (see for example, refs [1–7]). The process is conducted in CLIA and CAP certified laboratories. An RUO kit is also available.
